# Prevention in stroke - Current state, present gaps and probable next steps

**DOI:** 10.1186/s42466-026-00479-3

**Published:** 2026-04-22

**Authors:** Christian S. Thielscher, Felipe A. Montellano, Dorothee Saur, Agnes Flöel, Gabor C. Petzold, Karl Georg Haeusler

**Affiliations:** 1 Department of Vascular Neurology, University Hospital Bonn, Bonn, Germany; 2 German Center for Neurodegenerative Diseases (DZNE), Bonn, Germany; 3 Institute of Clinical Epidemiology and Biometry, University of Würzburg, Würzburg, Germany; 4 Department of Neurology, University Hospital Würzburg, Würzburg, Germany; 5 Department of Neurology, University of Leipzig Medical Center, Leipzig, Germany; 6 Department of Neurology, University Medicine Greifswald, Greifswald, Germany; 7 German Center for Neurodegenerative Diseases (DZNE), Rostock/Greifswald, Greifswald, Germany; 8 Department of Neurology, Universitätsklinikum Ulm, Ulm, Germany

## Abstract

**Background:**

Stroke is a leading cause of death and disability, and the top contributor to neurological disability-adjusted life years globally. Current trends suggest that stroke incidence will continue to rise, especially in low- and middle-income countries.

**Main body:**

Prevention opportunities are substantial because the majority of stroke burden is linked to modifiable risk factors. Thus, stroke incidence can effectively be reduced if proven interventions are implemented early, consistently, and at sufficient scale. Effective prevention requires both individualized, risk-based targets and interventions, and health-system approaches that support systematic detection, longitudinal control, and sustained adherence in routine care. Importantly, prevention benefits will be maximized only if access is equitable and if prevention goals can be achieved across the population at large, including so far underserved groups that combine multiple cardiovascular risk factors and low affinity to traditional health care prevention programs.

**Future directions:**

Next steps may include strengthening of primary-care-based prevention programs, routine surveillance of risk-factor control, outreach to underserved groups, and transparent quality indicators. Long-term follow-up is essential to sustain risk-factor control and to enable translation of evidence into population-level impact.

**Supplementary Information:**

The online version contains supplementary material available at 10.1186/s42466-026-00479-3.

## Introduction

According to the WHO definition, stroke is defined as “rapidly developing clinical signs of focal (or at times global) disturbance of cerebral function, lasting > 24 hours or leading to death, with no apparent cause other than vascular” [1]. Definitions that are more recent use a tissue-based approach that includes central nervous system infarction as well as intracerebral and subarachnoid hemorrhage, with or without clinical signs [2]. Of note, the updated WHO stroke definition now also includes events with symptoms lasting less than 24 hours as stroke (previously classified as Transient ischemic attack (TIA)), if ischemia is proven on neuroimaging [3]. Regional heterogeneity in stroke incidence and outcomes relates to socioeconomic differences, variation in exposure to metabolic, behavioral, and environmental stroke risk factors as well as uneven access to prevention and care [4]. These findings argue for coordinated prevention, care, and rehabilitation policies, particularly where resources and access are constrained [5].

In the following, we provide an overview regarding global stroke burden, stroke risk factors, staged stroke prevention and present gaps as well as strategies to improve stroke prevention. Given the complexity of the topic, we can only highlight selected aspects based on a selective literature search by the authors (for details, please refer to the Methods section in the Supplement). This paper was prepared within an initiative of the German Society of Neurology to strengthen prevention across different neurological diseases. The present review was written on behalf of the *German Society of Neurology’s Commission on Cerebrovascular Diseases*.

## Global burden of stroke

Neurological diseases are the leading cause of disability-adjusted life years (DALYs) worldwide, with stroke ranking first among them [6]. Globally, the Global Burden of Disease (GBD) Study estimated about 11.9 million incident strokes and 7.3 million stroke-related deaths for the year 2021, resulting in 160 million DALYs due to stroke, underlining the importance of stroke prevention. Besides stroke-related neurological deficits, stroke also impacts cognitive function and mental health, increasing the prevalence of depression, fatigue, pain, dementia and even suicide [7].

Ischemic stroke accounts for most incident strokes globally (~ 65%), whereas intracerebral hemorrhage (~ 29%) and subarachnoid hemorrhage (~ 6%) comprise a smaller share of events but contribute disproportionately to stroke-related DALYs due to higher mortality and long-term disability, [9, 10] particularly for ICH (about 80 million DALYs for intracerebral hemorrhage, 70 million for ischemic stroke) [8–10]. Notably, low- and middle-income countries account for 83% of strokes worldwide and 89% of stroke-related DALYs [8]. Projections suggest the share of low- and middle-income countries will even rise until 2050 [5]. Since the mid-2010s, global declines in age-standardized stroke rates - which had been the trend for the past decades - have stalled, with increases observed in Asia and Oceania, and among people younger than 70 years of age [8].

The lifetime risk at age 25 to suffer from stroke is about 25% at present, with ranges from 11% to 39% in different countries.[11] While stroke is still predominantly a disease of older adults, children can be affected [12], and younger adults account for a substantial proportion of events, as about 15% of all strokes occur in people aged 15–49 years. Between 2015 and 2021, age-standardized rates of ischemic stroke in this age group have risen.[13].

In Europe, projections suggest a modest increase in incident strokes (of 3%) until 2047, but an increased prevalence (+ 27%), accompanied by fewer stroke-related deaths (− 17%) and fewer DALYs (− 33%) - a pattern driven by aging populations but improved survival [14]. It is expected that the burden of ICH will rise marginally (+ 0.6%) in Europe up until 2050 [15]. Notably, a recent study in a western European population reported an increase in incidence of ischemic stroke by 18%, driven by episodes of TIA now being classified as stroke. In addition, this tissue-based definition is also likely to reduce case-fatality rate.[16].

Without intensified efforts of stroke prevention, the annual number of global deaths due to stroke is projected to increase by 50–70% until 2050, [5, 7] with 90% of deaths then occurring in low- and middle-income countries. Therefore, the global economic burden is projected to rise from US$ 890 billion in 2017 to US$2.3 trillion by 2050 [5]. This increase could be mitigated, at least in part, by intensified prevention (Table 1 for a general description of different levels of stroke prevention) [17].

**Table 1:** Levels of prevention in stroke across the life course

Level of prevention	Objective	Target population	Examples applied to stroke
Primordial	Prevent the emergence of risk factors by shifting social, environmental, and commercial determinants of health	Whole population or communities	Tobacco-control policies; WHO global sodium benchmarks that drive food reformulation; WHO Air Quality Guidelines (2021) for PM₂.₅
Primary	Prevent a first stroke by detecting and controlling established risk factors and promoting protective behaviors	Individuals without prior TIA or stroke	Detection and control of established risk factors, such as hypertension, dyslipidemia, diabetes, adiposity, tobacco and alcohol exposure
Secondary	Prevent recurrence of TIA or stroke by aggressive risk factor control plus etiology-specific strategies	Patients after TIA or stroke	As in primary prevention, additionally etiology-specific strategies (e.g., anticoagulation for atrial fibrillation, carotid revascularization where indicated)
Tertiary	Rehabilitation and longer-term care to reduce disability, prevent complications, and restore participation after stroke	Patients after stroke with residual deficits	Early, coordinated multidisciplinary rehabilitation; swallowing therapy; Screening and treatment of depression and cognitive impairment
Quaternary	Protect patients from over-medicalization, overdiagnosis, and harmful interventions	All patients, especially with multimorbidity or frailty	Caution against unnecessary screening procedures (e.g., screening asymptomatic adults for carotid stenosis)

The table summarizes the five complementary levels of prevention - primordial, primary, secondary, tertiary, and quaternary - linking each level of prevention to its core objective, target population, and illustrative examples relevant to stroke

### Stroke risk factors

Contemporary GBD estimates indicate that 23 modifiable risk factors accounted for more than 80% of global stroke burden in 2021, underscoring a large, addressable fraction of risk [4]. Within this framework, the leading metabolic contributors (Table 2) are high systolic blood pressure (consistently the largest driver across regions and especially prominent for intracerebral hemorrhage, contributing to ~ 55% of overall stroke DALYs in Western Europe), elevated LDL-cholesterol, high fasting plasma glucose, and high body mass index (BMI). Major behavioral risks (Table 3) include tobacco use, harmful alcohol use, unhealthy diet (e.g., high sodium and low fruits or vegetables), and physical inactivity. Among environmental risks (Table 4), ambient PM₂.₅ air pollution contributes substantially to stroke DALYs in many regions [8]. Very high or very low temperatures are also associated with higher stroke incidence and mortality, with subtype-specific patterns for ischemic versus hemorrhagic stroke [18]. Looking ahead, climate change is expected to amplify heat-associated stroke burden in many regions [19–21].

**Table 2:** Metabolic risk factors for stroke

Risk factor	Primordial	Primary	Secondary	IS risk rank	ICH risk rank	Knowledge gaps
High systolic blood pressure	Salt target or substitution; support of active mobility (walking, biking); labeling of products high in sodium	Systematic BP screening; home BP measurements; lifestyle bundle intervention; treat secondary causes; guideline-adherent medication	Tight control (< 130/80 mmHg); support adherence; manage hypotension	1; 55.8%	1; 55.4%	Individual BP targets; role of digital/wearable monitoring devices; pragmatic implementation trials; first line medication mix; role of polypill in diverse settings; effects of labeling on consumer attitudes
High LDL-cholesterol	Trans-fat regulation; marketing regulation on food; healthy food in public organizations; labeling of products high in trans-fat	Medication (statin, ezetimibe etc.) according to individual LDL targets; guideline-adherent medication	Intensive LDL lowering after IS; evaluation of add-on therapy	2; 32.4%	Not reported	Net benefit of low LDL in patients with high risk of bleeding; LDL targets after ICH; effects of PCSK9; Lp(a) lowering; effects of labeling on consumer attitudes
High fasting plasma glucose/diabetes	Healthy food environments; taxes on sugar-sweetened beverages; active mobility; labeling of products high in sugar	Screening for pre-diabetes; lifestyle interventions; weight management; guideline-adherent medication	Individualized glycemic targets; multifactorial control	3; 17.2%	9; 5.1%	GLP-1RA/SGLT2 for primary stroke prevention; weight-loss strategies, effects of labeling on consumer attitudes
Kidney dysfunction	Prevention via hypertension and diabetes control; nephrotoxin control, e.g. from polluted water	eGFR/albuminuria screening in risk groups; medication dose adjustment	Optimize cardiovascular risk factors; medication dose adjustment	6; 8.3%	5; 7.7%	SGLT2 effects on stroke outcomes in chronic kidney disease; volume management and medication dosing during heat waves
High body-mass index	Taxes and marketing limits on food and beverages; support of active mobility; childhood education	Lifestyle interventions, e.g. Planetary health diet; guideline-adherent medication; bariatric options; digital support programs	Weight programs in rehabilitation; prevention of sarcopenia	7; 7.3%	7; 5.5%	Long-term stroke outcomes of BMI-lowering medication; body composition markers besides BMI and waist-to-hip ratio

The table summarizes metabolic stroke risk factors as defined in the Global Burden of Disease (GBD) framework, alongside corresponding approaches for primordial, primary, and secondary prevention. IS risk rank and ICH risk rank indicate the relative rank order of attributable stroke burden (DALYs) for ischemic stroke (IS) and intracerebral hemorrhage (ICH) in Western Europe based on GBD 2021 estimates, and the table also reports the attributable percentage of stroke DALYs for each risk factor [8]. Not reported indicates that subtype-specific ranking was not available in the referenced GBD outputs. Key knowledge gaps relevant to prevention for each risk factor are outlined

Primary and secondary stroke prevention measures as well as present knowledge gaps of most promising prevention strategies are also listed in Tables 2, 3 and 4.

While its contribution is not systematically documented in the GBD, other stroke risk factors such as sleep-related disorders (e.g., sleep apnea [22], night-shift work [23]), acute infections [24], and chronic inflammation [25] likely remain insufficiently addressed so far. Notably, there are several risk scores used in clinical practice to roughly estimate the individual stroke risk based on the presence of specific risk factors (e.g., the ABCD2 score or the CHA_2_DS_2_-VASc score), impacting on medical treatment in primary (like the CHA_2_DS_2_-VASc score in atrial fibrillation) as well as secondary stroke prevention (e.g., ABCD2 score in TIA patients without a cardiac source of embolism; CHA_2_DS_2−_VASc score in atrial fibrillation) [26, 27].

**Table 3:** Behavioral risk factors for stroke

Risk factor	Primordial	Primary	Secondary	IS risk rank	ICH risk rank	Knowledge gaps
Alcohol use	Pricing/Taxation; availability controls; marketing limits	Screening; interventions, Alcohol use disorder treatment	Harm-reduction and relapse prevention	4; 11%	3; 11.7%	Causal impact of pricing/availability on stroke; digital follow-up models
Smoking (active)	Pricing/Taxation; ad bans; smoke-free laws	Cessation (counselling, nicotine replacement/varenicline/bupropion)	Intensified cessation and relapse prevention	5; 9.7%	2; 14.6%	Effects of e-cigarettes/heated tobacco; global taxation; best post-stroke delivery models
Low physical activity	Active transport; safe streets; parks/green spaces	≥ 150 min/week moderate-intensity activity (or equivalent)	Rehabilitation-adapted, supervised exercise	10; 4.4%	Not reported	Optimal dose/intensity post-stroke; durability of wearable-guided programs
Diet low in whole grains	Improve access/affordability; procurement	Dietitian-guided substitutions	Integration in post-stroke menus, e.g. in hospitals	11; 4.2%	Not reported	Substitution trials with stroke-specific endpoints
Diet high in sodium	Reformulation; sodium targets; clear labeling	Mediterranean diet; dietitian support	Post-stroke sodium restriction	12; 4.1%	10; 4.7%	Mandatory vs. voluntary reformulation; KCl salt-substitution at large scales; effects of labeling on consumer attitudes
Secondhand smoke	Comprehensive smoke-free enforcement	Exposure reduction in public spaces	Household cessation engagement	14; 1.9%	13; 2.8%	Enforcement – outcome studies
Diet high in processed meat	Food-system nudges; marketing/label controls; taxes	Diet swaps; limit processed meat; Planetary health diet	Heart-healthy diet coaching; Planetary health diet	15; 1.7%	Not reported	Stroke-specific effects; better subtype data; need for food supplementation; counseling methods
Diet low in fruits	Subsidies and affordability; institutional standards	Primary care counseling and recommendations, e.g. 5-a-day	Integration in post-stroke menus, e.g. in hospitals; address reduced appetite	16; 1.3%	8; 5.2%	Specific Planetary health diet recommendations; adherence with dysphagia
Diet high in red meat	Food-system nudges; marketing/label controls; taxes	Diet swaps; limit red meat; Planetary health diet	Heart-healthy diet coaching; Planetary health diet	16; 1.3%	Inverse correlation; −10.2%	Stroke-specific effects; better subtype data; need for food supplementation; counseling methods
Diet low in fiber	Food-environment improvements; reformulation targets	Fiber targets; Planetary health diet	Integrate into menus	18; 1.2%	12; 2.9%	Recurrence endpoints; scripts for counseling and dietitian referral
Diet low in vegetables	Subsidies and affordability; institutional standards	Primary care counseling and recommendations, e.g. 5-a-day	Integration in post-stroke menus, e.g. in hospitals	19; 1.1%	14; 0.1%	Specific Planetary health diet recommendations; adherence post-stroke
Diet high in sugar-sweetened beverages	Taxes; marketing limits; water availability	Swap to unsweetened drinks; track and reduce intake	Weight/metabolic support	20; 0.4%	Not reported	Effect of taxes on stroke and population subgroups
Diet low in polyunsaturated fatty acids (PUFA)	Policy with nutrition standards, e.g. for oils; procurement goals	Replace saturated fat with PUFA	Diet quality support	21; 0%	Not reported	Optimal PUFA sources and targets after stroke; ICH evidence

The table summarizes behavioral stroke risk factors as defined in the Global Burden of Disease (GBD) framework, alongside corresponding approaches for primordial, primary, and secondary prevention. IS risk rank and ICH risk rank indicate the relative rank order of attributable stroke burden (DALYs) for ischemic stroke (IS) and intracerebral hemorrhage (ICH) in Western Europe based on GBD 2021 estimates, and the table also reports the attributable percentage of stroke DALYs for each risk factor [8]. Not reported indicates that subtype-specific ranking was not available in the referenced GBD outputs. Key knowledge gaps relevant to prevention for each risk factor are also outlined

Although there are regional variations in the relative importance of most individual risk factors for stroke, ten potentially modifiable risk factors are associated with about 90% of the population-attributable risk of stroke in major regions of the world, as demonstrated in the INTERSTROKE study [28]. Adding on to these modifiable risk factors, non-modifiable risk factors (like age, sex, ancestry, and genetic predisposition) shape baseline risk and help refine and tailor prevention pathways (e.g., sex-specific profiles or risk scores for ischemic stroke). Older age raises absolute risk for stroke and other cardiovascular events at any given level of risk factors. Subsequently, the same relative treatment effect translates into larger absolute benefit in older adults, taking blood-pressure lowering as an example [29, 30]. At the same time, multimorbidity becomes more common with age and should guide the choice and intensity of pharmacologic and non-pharmacologic prevention in routine care [31]. Beyond that, factors such as sex, genetic background, and ancestry modulate both overall stroke rates and subtype distribution [32]. For example, several Asian populations carry a higher burden of intracerebral hemorrhage than Caucasian populations [8].

**Table 4:** Environmental risk factors for stroke

Risk factor	Primordial	Primary	Secondary	IS risk rank	ICH risk rank	Knowledge gaps
Ambient PM₂.₅ air pollution	Emission standards (e.g. transport/energy/heating); tight limits; urban green spaces; 15-minute-cities; promotion of e-mobility	Limit exposure; Exposure alerts in high-risk situations; indoor filtration	Limit exposure; Exposure alerts in high-risk situations; indoor filtration; exposure-reduction counseling	8; 6.7%	6; 6.8%	Individual exposure effects; Health effects of low-emission zones or public green spaces; high quality evidence of home filtration; personal exposure metrics
Low ambient temperature	Sufficient heating; cold-weather plans	Risk communication for vulnerable groups	Personalized cold action plans	9; 6.4%	4; 8.1%	Compound exposures (cold + PM₂.₅); interaction with specific medications; population at highest risk
Lead exposure	Lead-safe housing; industrial emission controls	Management in exposed individuals	Occupational health	12; 4.1%	11; 4%	Policy evaluation; clinical testing indications
Household air pollution (solid fuels)	Clean-fuel transition with support for low-income households	stove/ventilation upgrades	Home adaptations	21; 0%	16; 0%	Successful implementation policies at scale
High ambient temperature	City cooling; hot-weather plans	Risk communication for vulnerable groups; hydration; medication adaptation	Personalized heat action plans	21; 0%	14; 0.1%	Randomized controlled trials of heat-action in high-risk patients; influence of other weather metrics

The table summarizes environmental stroke risk factors as defined in the Global Burden of Disease (GBD) framework, alongside corresponding approaches for primordial, primary, and secondary prevention. IS risk rank and ICH risk rank indicate the relative rank order of attributable stroke burden (DALYs) for ischemic stroke (IS) and intracerebral hemorrhage (ICH) in Western Europe based on GBD 2021 estimates, and the table also reports the attributable percentage of stroke DALYs for each risk factor [8]. Not reported indicates that subtype-specific ranking was not available in the referenced GBD outputs. Key knowledge gaps relevant to prevention for each risk factor are also outlined

## Dimensions of stroke prevention

The stagnating stroke incidence rates in many regions and growing numbers of stroke survivors reinforce the need for intensified stroke prevention, equitable risk factor control, and consistent surveillance capable of tracking trends, gaps and successes across age strata and geographies.[5] To structure health action aimed at avoiding disease, prevention is often described as comprising five levels, from primordial through quaternary prevention (summarized in Table 1 also linking each level to core objectives, target population, and illustrative examples) [33].

***Primordial stroke prevention*** acts upstream to prevent the emergence of risk factors by shifting social, environmental, and commercial determinants of health [34], naming the WHO global sodium benchmarks that drive food reformulation and lower population blood pressure [35], and the WHO Air Quality Guidelines (2021) for PM₂.₅ [36] as examples.

***Primary stroke prevention*** targets people without prior cerebrovascular events but with established risk factors (Tables 2, 3 and 4), aiming to prevent a first stroke through detection and control of established risk factors [37]. Of note, patients without stroke who meet relevant healthy lifestyle criteria (i.e., no tobacco use, regular physical activity, healthy diet, normal weight) have a substantially reduced risk of cardiovascular diseases and stroke specifically compared to patients who do not meet any of these criteria [38, 39]. Besides the reduction of stroke burden, control of cardiovascular risk factors is supporting brain health and prevents dementia [40, 41].

***Secondary stroke prevention*** applies after TIA or stroke to prevent recurrence, combining intensive risk factor control (Tables 2, 3 and 4) and lifestyle modification with etiology-specific strategies (e.g., oral anticoagulation in atrial fibrillation, carotid revascularization in symptomatic carotid stenosis) [7, 42–45]. Importantly, many interventions are helpful in primary and secondary prevention, because they address the same underlying risks. To exemplify relative importance, we present the attributable distribution of stroke risk factors for Central Europe in Tables 2, 3 and 4, ordered by the magnitude of each risk factor’s contribution [8].

Given the breadth of pharmacological options for primary and secondary stroke prevention, a detailed discussion is outside the scope of this review. We therefore refer to current clinical practice guidelines, e.g. the European Stroke Organisation (ESO) guideline on pharmacological interventions for long-term secondary prevention after ischemic stroke or transient ischemic attack [37, 42–45], as well as the updated *Stroke Action Plan for Europe 2018–2030*, listing recommended diagnostic investigations in patients with suspected or confirmed stroke as well as secondary prevention interventions after stroke or TIA, stratified by stroke type and underlying etiology [7].

Although stroke risk factors and their treatment are well established, targets of secondary prevention are frequently not achieved in routine care. Structured follow-up beyond initial acute care and rehabilitation may help close this implementation gap. Trials of intensified, structured outpatient follow-up after stroke have demonstrated improved secondary prevention through better control of stroke risk factors with more intensive follow-up, but without a measurable reduction in recurrent vascular events within the respective observation periods [46, 47]. In addition, multimodal and psychological interventions can be used to improve patients’ knowledge about secondary prevention and to promote physical activity and healthy dietary habits [48].

***Tertiary stroke prevention*** comprises rehabilitation and longer-term care aimed at reducing disability, preventing complications, and restoring participation after stroke [49]. This includes coordinated multidisciplinary rehabilitation [50], language therapy [51], swallowing therapy [52], treatment of post-stroke depression and cognitive impairment [53], caregiver education [54], return-to-work support [55], and community reintegration [56].

***Quaternary prevention***, in turn, focuses on protecting patients from over-medicalization or harmful interventions. Consideration of quaternary prevention leads to caution against unnecessary screening procedures (e.g., screening asymptomatic adults for carotid stenosis [57]), initiating or extending therapies beyond guideline-supported indications (e.g., prescribing routine antiseizure prophylaxis after ischemic stroke [58] or unnecessarily prolonged dual antiplatelet therapy after ischemic stroke or TIA [44]).

While tertiary and quaternary prevention complete continuous stroke care, the bulk of risk reduction is achieved in primordial, primary, and secondary prevention.

### Socio-economic context of stroke prevention

Exposures to stroke risk factors and opportunities for prevention should be interpreted within their socio-economic context, which is shaped by politics, law, ethics, culture, social structure, and commercial factors. First, political priority-setting and governance, such as plans for noncommunicable diseases (NCD), including stroke, financing, and monitoring, define the capacity of prevention and determine whether proven measures can be implemented [59]. On that foundation, legal instruments translate intent into population-level change. In tobacco control, for example, the WHO Framework Convention on Tobacco Control and its MPOWER package have reduced smoking and second-hand smoke where comprehensively adopted [60]. To ensure those measures reach the right people, ethical commitments to equity must be embedded not only in service delivery but also in guideline conception - shaping which populations and outcomes are prioritized, how heterogeneity is analyzed, and which implementation strategies are recommended to close gaps in care [61].

Whether policies and services are accepted and effective then depends on anthropological and cultural contexts that shape diet, alcohol use, physical activity, and exposure to environmental risk factors. Thus, there is a need for culturally adapted interventions and empowerment of individuals [62]. These dynamics play out along social gradients in income, education, housing, and work, structuring differential exposure and access to prevention and care and thereby producing unequal health outcomes [63]. Finally, commercial actors, especially in the food, alcohol, tobacco, and fossil-fuel sectors, shape the defaults of availability, price, and promotion, and are responsible for substantial harm worldwide [64]. Taken together, this systematic perspective explains why similar individual behaviors yield very different population burdens across places and underlines why policy and market regulation must complement clinical care in order to reach implementation at scale. Prevention gains are maximized when services are organized to detect and control risk factors longitudinally and when access is equitable, particularly for underserved groups facing clustered risks and barriers to care. This requires accountability through measurable targets (e.g., blood-pressure control rates, door-to-needle times) [65], continuous surveillance, and quality indicators.

### Initiatives on stroke prevention

Building on the socio-economic context of prevention, many effective stroke prevention approaches explicitly modify and regulate upstream determinants (policy, commercial drivers, and environmental exposures), to strengthen service delivery and achieve measurable, system-level improvements. Accordingly, common features across initiatives worldwide include: packaging preventive measures into pragmatic bundles across the life course and across levels of prevention (primordial, primary, and secondary) and integrating stroke prevention into broader NCD and primary-care platforms. Equity is increasingly treated as a core performance domain, while digital health tools are leveraged to support risk assessment, longitudinal follow-up, patient education, adherence, and population-level surveillance. In parallel, upstream environmental prevention - particularly air quality and climate resilience - has gained prominence alongside classic levers such as tobacco control and salt reduction. (see Tables 3 and 4)

At the global level of prevention, specific examples of initiatives include the WHO *“Intersectoral Global Action Plan on Epilepsy and Other Neurological Disorders (2022–2031)*”, which outlines governance priorities and strengthening of health services for neurological disorders [66], and the WHO “*NCD Best Buys*” which prioritize high-yield population policies targeting tobacco, alcohol, diet, and physical inactivity [67], also mentioning the cornerstones of acute stroke care (stroke unit treatment, recanalization therapies like thrombolysis and mechanical thrombectomy) [68]. These WHO initiatives are complemented by practical primary-care implementation toolkits such as “*HEARTS”* to operationalize cardiovascular risk management and improve risk factor detection and control [69]. Stroke-specific guidance and advocacy are advanced globally by the World Stroke Organization through service recommendations that span the full pathway - from pre-hospital triage and stroke unit coverage to rehabilitation and long-term support [70, 71] - alongside a patient-rights-based agenda for equitable access to care (*"Global Stroke Bill of Rights"*) [72]. Regional examples of large-scale initiatives on cardiovascular and more specifically stroke prevention include the European “*Healthier Together*” program, which supports scaling best practices with an explicit focus on social determinants and equity [73], while the European Stroke Organization “*Stroke Action Plan*”, initiated in 2019 in cooperation with the *Stroke Alliance for Europe* and recently updated, covers the entire chain of stroke care and sets measurable stroke-specific targets with an overall goal of reducing incident strokes by > 15% by 2030 [7, 74]. According to the *Stroke Action Plan* authors, “gaps remain across the care pathway but particularly in terms of access to stroke units, rehabilitation and follow-up”. The following issues were named as key to reduce the burden of stroke in Europe: (1) “national stroke plans, […] reflected in the reimbursement systems”, (2) control of quality and outcome on the individual level and in the health care system; (3) “equal access to sustainable, timely and evidence-based stroke care” and (4) “effective national strategies to promote and facilitate a healthy lifestyle and risk factor control” [7].

In the US, “*Million Hearts 2027*” concentrates on a small set of high-yield priorities with an explicit equity focus, having the aim of averting 1 million cardiovascular events in five years [75]. At the same time, US initiatives such as “*Get With The Guidelines-Stroke*” use registry-based quality improvement to close care gaps [76]. Additional examples include the Gramado Declaration as a regional commitment in Latin America, focusing on national stroke policies, service expansion, public awareness, and access to thrombectomy and telemedicine [77], and China’s National Stroke Prevention and Control Program [78], combining population risk factor policies, risk-based community screening with a referral ladder and registry-based performance and outcomes measures [79].

For a more detailed description of selected global initiatives, please refer to the *Supplementary Material*.

### The need for policy measures in stroke prevention

**Fig. 1:**
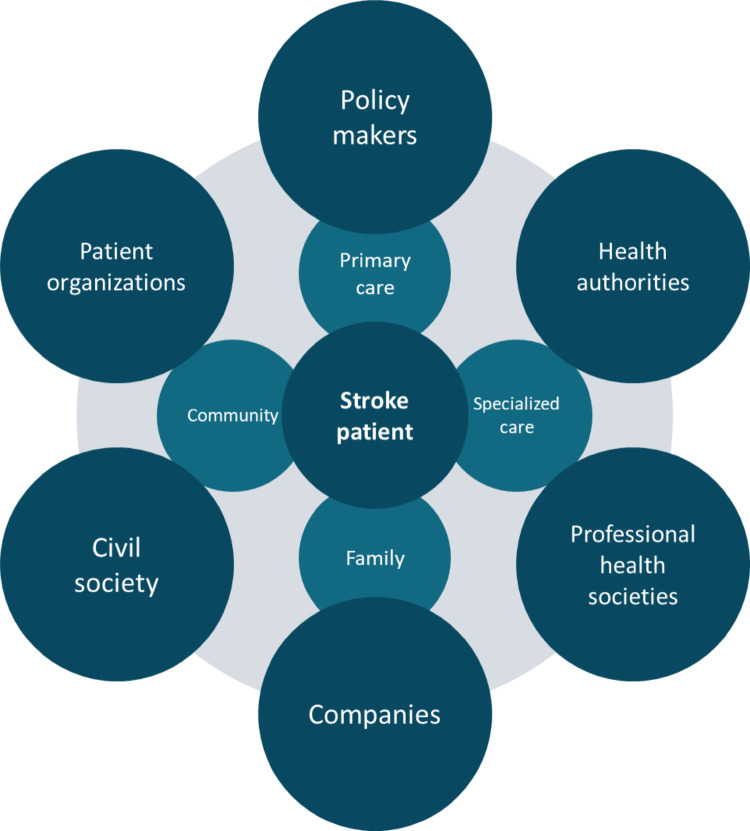
Multi-stakeholder ecosystem for secondary and tertiary stroke prevention. The stroke patient is positioned at the center. The inner tier represents stakeholders with direct, day-to-day influence on individual prevention and risk factor management. The outer tier depicts system-level actors that shape the structural conditions for effective prevention, including regulation, financing, health service organization, advocacy, and social determinants of health

Preventing stroke requires coordinated action across society, and prevention efforts rarely achieve the desired effectiveness without political and organizational levers (Fig. 1). Policymakers and legislators must enact and enforce fiscal and regulatory measures and secure sustained financing. Moreover, health authorities need to scale primary-care-based risk assessment and management and should work toward equitable access to prevention, acute care, rehabilitation, and community reintegration. Municipal authorities and urban and environmental planners can create healthier, cleaner environments that lower stroke risk, including simple measures such as opportunities for walking and biking in everyday life within cities [80]. Stroke risk is also closely intertwined with climate action, also impacting stroke risk [19, 81]. Moreover, poverty reduction should be achieved, as poverty also elevates stroke risk [82]. Civil society, patient organizations, and both traditional and digital media are vital for awareness and health literacy and can help to enable and tailor patient-specific prevention.

Most of the aforementioned measures and especially scaling up prevention require investment, but enhanced health services are likely to deliver economic returns [83]. Notably, a prioritized set of policies is needed to shape stroke prevention packages, as cardiovascular risk profiles differ across countries. Therefore, programs should be context-specific, focusing on interventions with the greatest impact, best cost-effectiveness, and practical feasibility.

Even where the benefits of policy measures are well established, implementation remains insufficient worldwide. As an example, global reductions in tobacco use have been achieved in line with the WHO Framework Convention on Tobacco Control (FCTC), but progress has slowed [84]. Where prevention deficits persist, they tend to cluster around sustainable financing, full pathway coverage (from primary prevention to community reintegration), supply chains for essential medicines, and the translation of high-level strategies into concrete national and subnational plans [66, 88]. Where such plans are absent, WHO toolkits[69] offer a practical framework for setting up or strengthening essential services.

International cooperation alongside national actions is needed to ensure effectiveness of present measures [85, 86]. Notably, many low-income countries lack the resources to implement (stroke) prevention proposals and must rely, in part, on external support [87, 88].

Implementation should be grounded in high-quality evidence. Where such studies exist, they often depend on national or international funders [89]. Funding may be raised through health-promoting taxes. Philanthropic actors also contribute substantially; however, many high-income countries allocate only a small share of development cooperation funding for health and often lack explicit policies [90].

### Translating evidence into action

Researchers should propagate translational stroke research, generate context-specific evidence and maintain high-quality registries and thus lay the foundation for future prevention initiatives [7]. Clinicians and health care teams must deliver evidence-based interventions and continuously measure implementation. Neurologists and other health care professionals can shape stroke prevention at the bedside and across borders, as a more equitable global prevention agenda is needed [91]. That includes partnership of health care professionals and institutions in high- as well as low- and middle-income countries to co-design registries, and multicenter trials with open methods and shared data standards; aiming for bidirectional training, mentorship, and telemedicine networks. Clinicians can advocate within their health systems for fair pricing of essential preventive drugs, invest in interoperable data infrastructure, and back community-led education that is linguistically and culturally tailored. This is especially critical for underserved populations and communities that have been inadequately reached by prevention efforts and receive insufficient attention in health system planning.

### Future directions of stroke prevention

A major area of research is a more precise, individualized assessment of stroke risk (beyond traditional clinical scores) enabling ***individualized stroke prevention***. In this regard, increasing attention is being paid to novel biomarkers [92] and non-invasive approaches that can capture vascular pathologies earlier and with greater accuracy and granularity [93]. Genetic information - such as polygenic risk profiles - could ultimately enable prevention to start earlier and to be tailored more specifically to stroke subtypes, provided that these models are robustly developed and validated across diverse ancestries [94]. Beyond risk stratification, prevention is increasingly framed as target-driven precision care: LDL cholesterol, blood pressure, and other cardiovascular risk factors should be aligned more closely with an individual’s overall risk rather than fixed thresholds [95].

***Digital technologies*** such as ***artificial intelligence*** (AI) can help translate this concept into practice by enabling patient-specific monitoring and feedback and by supporting implementation - such as improving medication adherence, encouraging physical activity, or facilitating smoking cessation. However, while digital interventions in primary prevention can deliver measurable gains in lifestyle and adherence outcomes, robust evidence for hard clinical endpoints is still limited so far, especially for incident or recurrent stroke [96]. Several proposed applications therefore remain hypothesis-generating and require pragmatic endpoint trials. Nevertheless, the growing use of wearables and other sensors offers the opportunity to capture risk factors far more continuously, and in ways that better reflect everyday life, than occasional clinic visits. Turning this data into actionable information, however, requires reliable methods for aggregation, quality control, and interpretation. Here, AI is often seen as the key enabler that can potentially transform large, heterogeneous datasets into individualized risk profiles and practical recommendations. A plausible future scenario is that adverse trends, such as a gradual loss of blood pressure control, declining physical activity, or changes in sleep patterns, are detected early. This could allow prevention to be initiated or intensified proactively, including individuals outside classical high-risk groups who are typically monitored less closely. From a population-health perspective, AI could also support system-level stewardship by using routine digital data to identify prevention gaps and underserved groups in the future [95]. Within this deployment of AI, large language models are increasingly discussed as an interface layer that could translate complex information into patient-friendly guidance and help sustain behavior change, adherence, and follow-up. As health data become more digitized and interoperable, recommendations could become more context-rich - for example by incorporating laboratory results, ECG (electrocardiogram) findings, or imaging data. At the same time, widespread adoption will require rigorous evaluation of performance and safety across real-world settings with noisy data of variable quality, and across diverse populations [95]. AI may also enable more targeted medication choices by predicting response or expected benefit [95]. For promoting physical activity, adaptive training plans that leverage wearable-derived parameters may make genuinely personalized activity programs more achievable [95]. Digital technologies could also broaden access to screening for and increasing the ability of detecting conditions associated with an elevated stroke risk, such as atrial fibrillation or sleep-related breathing disorders, also refining selection of patients at high-risk of stroke [97, 98]. To deliver meaningful preventive benefit, such tools must be embedded in local care pathways. Training datasets should be sufficiently diverse to reduce bias, and issues around data acquisition and data security need to be addressed adequately [99]. To ensure that digital health applications are effective and useful in routine care, health care professionals should be actively involved in design, target definition, and evaluation so that digital prevention is developed and assessed against outcomes that matter [100].

***Genetic research*** could reshape stroke prevention in several ways. Genome-wide Association Studies (GWAS)-based approaches may ultimately yield subtype-specific genetic risk profiles, but this will require broader coverage and validation across ancestries to minimize bias and ensure generalizability [94]. Looking ahead, genetics may also help predict not only incident risk but also post-stroke outcomes and complication risk, thereby further individualizing secondary prevention [101]. Finally, genetics provides a route to more rational drug development: genetically supported causal inference can help prioritize targets and streamline the research pipeline for novel preventive therapies [94].

Even though established pharmacotherapies already cover a large share of effective stroke prevention, ***novel (non-)pharmacological strategies for primary and secondary prevention*** may further improve the benefit–risk balance and address residual risk more precisely. As an example, left atrial appendage occlusion is being discussed as an option to avoid long-term anticoagulation in atrial fibrillation patients with ICH, and may add to ischemic stroke prevention on top of oral anticoagulation in others [102]. Beyond this, multiple agents are in development that seek to reduce stroke risk without increasing bleeding. Factor XI inhibitors are a prominent example; while they appear safe, evidence for meaningful stroke reduction remains inconclusive so far, and is being evaluated in ongoing phase III trials [103]. In lipid management, several drug classes now enable highly effective LDL lowering. Increasingly, however, attention is directed at other components of lipid metabolism risk, including triglycerides and lipoprotein(a), which are epidemiologically important drivers of cardiovascular risk but remain less readily modifiable. Emerging targets could support a more holistic, individualized lipid risk profile with more differentiated therapeutic options - provided that ongoing outcome trials demonstrate clinical benefit [104]. Inflammation is increasingly viewed as another potential target in cardiovascular prevention. Biomarkers such as C-reactive protein and Interleukin-6 (IL-6) are associated with elevated stroke risk and could be incorporated into risk models and the tailoring of primary and secondary prevention strategies [25]. To date, however, anti-inflammatory therapies such as colchicine have not shown consistent benefit on clinical endpoints after stroke (in contrast to coronary artery disease) [105]. This may indicate that more precise identification of patients most likely to benefit, particularly in secondary prevention, is required. Additional anti-inflammatory agents, including IL-6-targeted therapies, are in development, and the coming years will show whether inflammatory pathways can be addressed reliably to reduce recurrent stroke risk [25].

Alongside precision approaches, implementation innovation remains an important pathway for future progress. The ***polypill*** combines multiple drug classes - most commonly antihypertensives and a statin, sometimes supplemented by antiplatelet therapy or other components - to address several risk factors simultaneously. This strategy may thereby improve adherence, scalability, and reach [106]. Rather than aiming for highly individualized therapy, this approach targets robust population-level risk reduction and may broaden access to primary and secondary prevention in low- and middle-income countries. Across a range of contexts, improvements in adherence and cardiovascular outcomes (including stroke) have been reported. However, more robust evidence is still needed for secondary prevention after stroke to refine target populations, polypill composition, and implementation strategies [107].

Across all innovations, it remains essential that new approaches demonstrate clinically meaningful benefits in the **real-world care context **[100]. At the same time, innovations in prevention must be designed to avoid creating additional barriers to access [108]. In ***epidemiological studies***, a consistent stroke case definition that also captures evidence of neuronal cell death could be helpful. Furthermore, future epidemiological studies should continuously capture metabolic, behavioral, and environmental risk factors using standardized, granular measurements. This enables country- and region-specific analyses and thus highest-yield targets for prevention [28].

## Summary

Although most risk factors for both ischemic stroke and intracerebral hemorrhage are well established and well documented, substantial gaps remain across primordial, primary, and secondary stroke prevention. At the same time, existing gaps represent the opportunity to reduce stroke incidence as well as stroke-related mortality and morbidity. Achieving this aim will require targeted, region-specific action by policymakers, society, and health care professionals. To make stroke prevention globally equitable, such efforts should be coordinated internationally and strengthened through sustained collaboration. This sets the stage for a future in which stroke prevention becomes both more comprehensive in reach and more personalized in delivery.

## Supplementary Information

Below is the link to the electronic supplementary material.

Supplementary Material 1

## Data Availability

Not applicable. No new datasets were generated or analyzed for this review.

## Abbreviations

AHA: American heart association
AI: Artificial intelligence
ASA: American stroke association
BMI: Body mass index
BP: Blood pressure
CVD: Cardiovascular disease
DALY(s): Disability-adjusted life year(s)
ECG: Electrocardiogram
eGFR: Estimated glomerular filtration rate
ESO: European stroke organisation
EU: European union
GBD: Global burden of disease
GLP-1RA: Glucagon-like peptide-1 receptor agonist
HEARTS: WHO HEARTS technical package for cardiovascular disease management in primary health care
ICH: Intracerebral hemorrhage
IL-6: Interleukin-6
IS: Ischemic stroke
LDL: Low-density lipoprotein (cholesterol)
Lp(a): Lipoprotein(a)
NCD(s): Noncommunicable disease(s)
PCSK9: Proprotein convertase subtilisin/kexin type 9
PM₂.₅: Fine particulate matter with aerodynamic diameter≤2.5 µm
PUFA: Polyunsaturated fatty acids
SAP-E: Stroke action plan for Europe
SGLT2: Sodium–glucose cotransporter 2 (inhibitor)
TIA: Transient ischemic attack
WHO: World health organization
WSO: World stroke organization
