# Supplementary material for: Prevention in stroke - Current state, present gaps and probable next steps
Source: Neurol Res Pract. 2026 Apr 22;8(1):31. doi: 10.1186/s42466-026-00479-3 (PMC13101267; doi:10.1186/s42466-026-00479-3)
Supplement: Supplementary file 1 — Supplementary Material 1 [file 42466_2026_479_MOESM1_ESM.docx]

**Supplementary Material**

**Methods**

In brief, this narrative review is based on a selective literature search in PubMed and OpenEvidence. The primary search term was “stroke prevention”, combined with section-specific terms as appropriate (e.g., stroke epidemiology, GBD, primordial, primary, secondary, tertiary, quaternary, risk factors, global, implementation, equity, digital health, polypill). Searches were restricted to English-language publications and prioritized large cohort studies, randomized trials, meta-analyses, reviews, and contemporary guideline documents and policy statements, particularly from the World Health Organization, national governments, the European Union, and scientific societies. We considered literature up to January 2026 and primarily focused on contemporary publications from 2020 onwards; however, earlier landmark publications were included where they remain foundational for definitions, prevention frameworks, or key concepts. As this is a narrative review, no formal systematic review procedures (e.g., PRISMA flow diagram) or structured risk-of-bias assessment were performed.

**Acknowledgements**

The authors would like to acknowledge the assistance of OpenAI’s ChatGPT-5.1 and ChatGPT-5.2 in editing and improving the linguistic clarity of this manuscript. All suggested edits were reviewed and approved by the authors, who take full responsibility for the content of this review.

**More detailed description of** **initiatives on stroke prevention**

This section provides a more detailed overview of some global initiatives for stroke prevention that were already briefly introduced in the main article.

On a global level, the *Intersectoral Global Action Plan on Epilepsy and Other Neurological Disorders* (2022–2031) outlines strategic targets for governance priorities, effective health services (including prevention, treatment and care), innovative and helpful research, and equal rights for those living with neurological disorders.^1^

The WHO “*Best Buys*” for noncommunicable diseases recommend more detailed and specific cost-effective general prevention policies (to reduce tobacco use, harmful use of alcohol, unhealthy diet and physical inactivity).^2^ More practical guidance can be derived from WHO technical packages such as *HEARTS*, which assists in cardiovascular risk management in primary care by offering best practice to providers across different levels of health care systems. E.g., healthy-lifestyle counseling is operationalized with easy-to-follow steps for implementation in primary care.^3^ In some regions, this approach is adopted and rolled out at a large scale, e.g. in the Americas.^4^ Stroke-specific resources complement these measures by the WHO.

The World Stroke Organization’s (WSO) “*Service Guidelines and Action Plan*” describe how to organize pre-hospital triage, stroke units, reperfusion, rehabilitation, and long-term support across different resource settings,^5^ the implementation details are described in roadmaps for local use.^6^ Adding on to that, the Global Stroke Bill of Rights emphasizes patients’ rights to optimal care and support. It encourages further advocacy on these issues.^7^

These global plans and frameworks underscore the need for combined political and organizational efforts in prevention, acute stroke care, and rehabilitation, and they offer guidance at multiple levels of implementation. Their impact depends on converting them into detailed plans adapted to local and regional contexts.

Several regional programs show different pathways to impact, many explicitly referencing WHO or WSO initiatives and programs, some are presented below as illustrative examples:

The European “*Healthier Together*” initiative guides EU4Health funding and collaboration into joint actions that scale best practices in cardiovascular prevention and care across member states, with an explicit focus on social determinants of health and equity.**^8^** Adding on to that, the Council Conclusions on Cardiovascular Health emphasize clear priorities for enhanced prevention, early detection, treatment, and rehabilitation, with the aim of developing a comprehensive EU cardiovascular health plan.^9^

Focusing on stroke in Europe, the *Stroke Action Plan (2018–2030)* of the European Stroke Organization provides measurable targets, such as intensified prevention with an overall goal of reducing incident strokes by 15%, comprehensive stroke unit coverage, national implementation plans, and research priorities.^10^,^11^ Regular reports are published to assess progress and to highlight implementation gaps.^12^

The UK’s “*Integrated Stroke Delivery Networks*” coordinate prevention, pre-hospital care, hyperacute and acute treatment (e.g. implementing standardized imaging and reperfusion pathways), rehabilitation, and post-discharge ‘life after stroke’ support under one national model.^13^ Progress to the targeted goals is ongoing but incomplete.^14^

In the United States, national efforts pair population-level prevention with data-driven quality improvement. “Million Hearts 2027**”** concentrates on a small set of high-yield priorities with an explicit equity focus, having the aim of averting 1 million cardiovascular events in five years.^15^ For more than two decades, “*Get With The Guidelines-Stroke*” has combined a national registry of real-world care with structured quality-improvement initiatives to promote timely, evidence-based stroke treatment at scale. In many US settings, stroke-center certification is linked to the submission of standardized performance measures to GWTG–Stroke or equivalent registries, ensuring continuous data capture and feedback. Leveraging these data, targeted programs have been launched to close key care gaps, e.g. “*Target: Stroke”*, which introduced stepwise goals to reduce door-to-needle times.^16^ Nonetheless, disparities, e.g. by race and ethnicity, persist.^17^

In Latin America, the *2018 Gramado Declaration* set multilateral commitments to draft national stroke policies, expand stroke centers, promote public awareness and risk-factor control, and pave the way for thrombectomy and telemedicine integration.^18^ Within two years, more public campaigns, higher number of stroke centers, and broader participation in common indicator registries were reported.^19^ Standardized packages such as *HEARTS* can improve prevention coverage, but implementation of prevention targets varies widely across Latin America and is shaped by national plans and guidelines, health-system financing, and resource availability, with notable disparities in access between publicly funded and private services.^20^

China has pursued a multi-pillar stroke-prevention strategy under *Healthy China 2030*^21^ and the National Stroke Prevention and Control Program (CSPPC),^22^ combining population risk-factor policies (e.g. salt reduction^23^), risk-based community screening with a referral ladder and registry-based performance and outcomes measures.^24^ Translating frameworks into uniform primary and secondary prevention coverage across provinces is still a work in progress, e.g. shown high geographic variation in stroke burden.^25^

Across these efforts, common success factors emerge: integrating stroke prevention within broader noncommunicable disease and primary-care platforms and initiatives;^26^,^27^ establishing stroke-focused service networks that are adapted to local needs^28^ and span the entire health system; developing and using performance indicators and registries to measure stroke-care quality at scale and identify the most relevant priority action areas;^29^ and an emphasis on ensuring equal access to services for all, with particular attention to women, minority groups, and migrants.^30^,^31^ Digital solutions can be leveraged to advance these objectives and provide patient-centered education and care offerings.^32^

References Supplementary Material

1. World Health Organization. Intersectoral global action plan on epilepsy and other neurological disorders 2022–2031. [License: CC BY-NC-SA 3.0 IGO]. Accessed October 1, 2025. https://www.who.int/publications/i/item/9789240076624

2. World Health Organization. Tackling NCDs: best buys and other recommended interventions for the prevention and control of noncommunicable diseases. [License: CC BY-NC-SA 3.0 IGO]. Accessed October 1, 2025. https://www.who.int/publications/i/item/9789240091078

3. World Health Organization. HEARTS technical package for cardiovascular disease management in primary health care: risk based CVD management. [License: CC BY-NC-SA 3.0 IGO]. Accessed October 1, 2025. https://www.who.int/publications/i/item/9789240001367

4. Ordunez P, Lombardi C, Picone DS, et al. HEARTS in the Americas: a global example of using clinically validated automated blood pressure devices in cardiovascular disease prevention and management in primary health care settings. *J Hum Hypertens*. 2023;37(2):126-129. doi:10.1038/s41371-022-00659-z

5. Lindsay P, Furie KL, Davis SM, Donnan GA, Norrving B. World Stroke Organization global stroke services guidelines and action plan. *Int J Stroke*. 2014;9 Suppl A100:4-13. doi:10.1111/ijs.12371

6. Lindsay MP, Norrving B, Furie KL, Donnan G, Langhorne P, Davis S. Global Stroke Guidelines and Action Plan: A Road Map for Quality Stroke Care. Roadmap Implementation Guide. Accessed February 2, 2026. https://www.world-stroke.org/assets/downloads/Global_Stroke_Guidelines_and_Action_Plan_All_in_one_English.pdf

7. World Stroke Organization. Global Stroke Bill of Rights Toolkit. Accessed October 1, 2025. https://www.world-stroke.org/assets/CVs/Global_Stroke_Bill_of_Rights_ToolKit_Brochure_2020_WEB.pdf

8. European Commission. Healthier Together: EU Non-Communicable Diseases Initiative. Accessed October 1, 2025. https://health.ec.europa.eu/system/files/2022-06/eu-ncd-initiative_publication_en_0.pdf

9. Council of the European Union. Conclusions on the Improvement of Cardiovascular Health in the European Union (ST-15315-2024-INIT). Accessed October 1, 2025. https://data.consilium.europa.eu/doc/document/ST-15315-2024-INIT/en/pdf

10. Norrving B, Barrick J, Davalos A, et al. Action Plan for Stroke in Europe 2018-2030. *Eur Stroke J*. 2018;3(4):309-336. doi:10.1177/2396987318808719

11. Christensen H, Pezzella FR, Roaldsen MB, et al. Stroke Action Plan for Europe 2018-2030 (SAP-E): mid-term review and update. *Eur Stroke J*. 2026;11(1). doi:10.1093/esj/aakaf026

12. European Stroke Organisation. Stroke Alliance for Europe. SAP-E Implementation Plan: 2023 Status Report. Accessed October 1, 2025. https://actionplan.eso-stroke.org/wp-content/uploads/2023/09/SAP-E-status-report_2023__Final-2.pdf

13. NHS England. National Stroke Service Model. Accessed October 1, 2025. https://www.england.nhs.uk/wp-content/uploads/2021/05/stroke-service-model-may-2021.pdf

14. Sentinel Stroke National Audit Programme. State of the Nation Report 2024: Stroke care received between April 2023 and March 2024. Accessed October 1, 2025.

15. Centers for Disease Control and Prevention. Million Hearts. About Million Hearts 2027. Accessed October 8, 2025. https://millionhearts.hhs.gov/about-million-hearts/index.html

16. Reeves MJ, Fonarow GC, Smith EE, Sheth KN, Messe SR, Schwamm LH. Twenty Years of Get With The Guidelines-Stroke: Celebrating Past Successes, Lessons Learned, and Future Challenges. *Stroke*. 2024;55(6):1689-1698. doi:10.1161/STROKEAHA.124.046527

17. Man S, Solomon N, Mac Grory B, et al. Trends in Stroke Thrombolysis Care Metrics and Outcomes by Race and Ethnicity, 2003-2021. *JAMA Netw Open*. 2024;7(2):e2352927. doi:10.1001/jamanetworkopen.2023.52927

18. Ouriques Martins SC, Sacks C, Hacke W, et al. Priorities to reduce the burden of stroke in Latin American countries. *Lancet Neurol*. 2019;18(7):674-683. doi:10.1016/S1474-4422(19)30068-7

19. Martins SCO, Lavados P, Secchi TL, et al. Fighting Against Stroke in Latin America: A Joint Effort of Medical Professional Societies and Governments. *Front Neurol*. 2021;12:743732. doi:10.3389/fneur.2021.743732

20. Ordunez P, Campbell NRC, DiPette DJ, et al. HEARTS in the Americas: Targeting Health System Change to Improve Population Hypertension Control. *Curr Hypertens Rep*. 2024;26(4):141-156. doi:10.1007/s11906-023-01286-w

21. Chen P, Li F, Harmer P. Healthy China 2030: moving from blueprint to action with a new focus on public health. *Lancet Public Health*. 2019;4(9):e447. doi:10.1016/S2468-2667(19)30160-4

22. Chao B-H, Yan F, Hua Y, et al. Stroke prevention and control system in China: CSPPC-Stroke Program. *Int J Stroke*. 2021;16(3):265-272. doi:10.1177/1747493020913557

23. Neal B, Wu Y, Feng X, et al. Effect of Salt Substitution on Cardiovascular Events and Death. *N Engl J Med*. 2021;385(12):1067-1077. doi:10.1056/NEJMoa2105675

24. Wang Y, Li Z, Wang Y, et al. Chinese Stroke Center Alliance: a national effort to improve healthcare quality for acute stroke and transient ischaemic attack: rationale, design and preliminary findings. *Stroke Vasc Neurol*. 2018;3(4):256-262. doi:10.1136/svn-2018-000154

25. Ma Q, Li R, Wang L, et al. Temporal trend and attributable risk factors of stroke burden in China, 1990-2019: an analysis for the Global Burden of Disease Study 2019. *Lancet Public Health*. 2021;6(12):e897-e906. doi:10.1016/S2468-2667(21)00228-0

26. Schwalm J-D, McCready T, Lopez-Jaramillo P, et al. A community-based comprehensive intervention to reduce cardiovascular risk in hypertension (HOPE 4): a cluster-randomised controlled trial. *Lancet*. 2019;394(10205):1231-1242. doi:10.1016/S0140-6736(19)31949-X

27. McGuire H, Van TB, Le Thi Thu H, et al. Improving hypertension awareness and management in Vietnam through a community-based model. *Sci Rep*. 2022;12(1):19860. doi:10.1038/s41598-022-22546-w

28. Singh A, Jenkins C, Calys-Tagoe B, et al. Stroke Investigative Research and Education Network: Public Outreach and Engagement. *J Community Med Health Educ*. 2017;7(2). doi:10.4172/2161-0711.1000518

29. Quiambao A, Malekpour M-R, Golestani A, et al. World health Organization's guidance for tracking non-communicable diseases towards sustainable development goals 3.4: an initiative for facility-based monitoring. *EClinicalMedicine*. 2025;85:103304. doi:10.1016/j.eclinm.2025.103304

30. Anand SS, Kandasamy S, Marchand M, et al. Reducing inequalities in cardiovascular disease: focus on marginalized populations considering ethnicity and race. *Lancet Reg Health Eur*. 2025;56:101371. doi:10.1016/j.lanepe.2025.101371.

31. Boden-Albala B. Roadmap for Health Equity: Understanding the Importance of Community-Engaged Research. *Stroke*. 2025;56(1):239-250. doi:10.1161/STROKEAHA.124.046958

32. Katz ME, Mszar R, Grimshaw AA, et al. Digital Health Interventions for Hypertension Management in US Populations Experiencing Health Disparities: A Systematic Review and Meta-Analysis. *JAMA Netw Open*. 2024;7(2):e2356070. doi:10.1001/jamanetworkopen.2023.56070
